# Porous Graphene Microflowers for High-Performance Microwave Absorption

**DOI:** 10.1007/s40820-017-0179-8

**Published:** 2017-12-21

**Authors:** Chen Chen, Jiabin Xi, Erzhen Zhou, Li Peng, Zichen Chen, Chao Gao

**Affiliations:** 1 MOE Key Laboratory of Macromolecular Synthesis and Functionalization, Department of Polymer Science and Engineering, Key Laboratory of Adsorption and Separation Materials & Technologies of Zhejiang Province, Zhejiang University, 38 Zheda Road, Hangzhou, 310027 People’s Republic of China; 2 Department of Mechanical Engineering, Zhejiang University, 38 Zheda Road, Hangzhou, 310027 People’s Republic of China

**Keywords:** Graphene, Microflowers, Porous, Microwave absorption

## Abstract

Graphene has shown great potential in microwave absorption (MA) owing to its high surface area, low density, tunable electrical conductivity and good chemical stability. To fully realize grapheneʼs MA ability, the microstructure of graphene should be carefully addressed. Here we prepared graphene microflowers (Gmfs) with highly porous structure for high-performance MA filler material. The efficient absorption bandwidth (reflection loss ≤ −10 dB) reaches 5.59 GHz and the minimum reflection loss is up to −42.9 dB, showing significant increment compared with stacked graphene. Such performance is higher than most graphene-based materials in the literature. Besides, the low filling content (10 wt%) and low density (40–50 mg cm^−3^) are beneficial for the practical applications. Without compounding with magnetic materials or conductive polymers, Gmfs show outstanding MA performance with the aid of rational microstructure design. Furthermore, Gmfs exhibit advantages in facile processibility and large-scale production compared with other porous graphene materials including aerogels and foams.

**Electronic supplementary material:**

The online version of this article (10.1007/s40820-017-0179-8) contains supplementary material, which is available to authorized users.

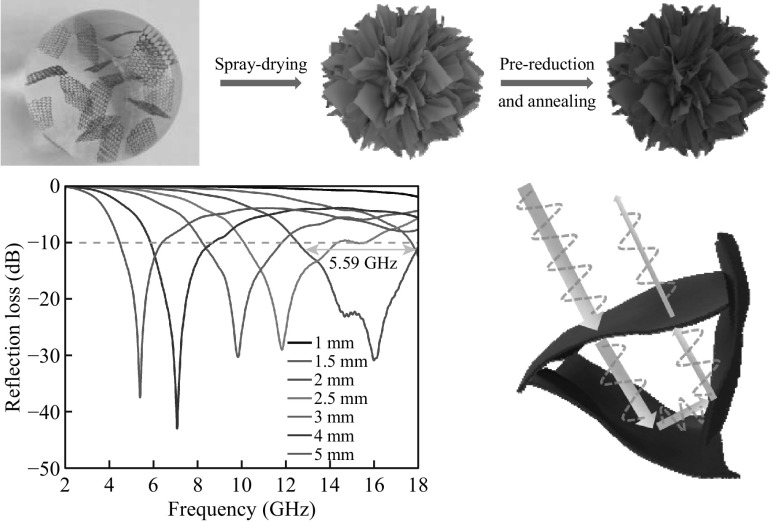

## Highlights

- Graphene microflowers (Gmfs) for outstanding microwave absorption performance are produced via a three-step protocol.
- The porous Gmfs show a broad efficient absorption bandwidth of 5.59 GHz with a minimum reflection loss of −42.9 dB, outperforming most graphene-based materials ever reported.
- The mass productivity, low filler content (10%) and low density (40–50 mg cm^−3^) of Gmfs are favorable for their practical applications.

## Introduction

Microwave technology is under rapid development since last century, covering extensive application areas, such as satellite communications, radar detections, information security and microwave heating [1, 2]. For the consideration of noise reduction and stealth technology, microwave absorption (MA) has received tremendous attention. Besides, physical protection against microwave promotes the development of MA technology [3]. In order to satisfy the requirements of low density, long duration and broad absorption bandwidth, ideal absorbers should possess several features: rational chemical composition for impedance match, low density, low filling ratio, cost efficiency, high thermal and chemical duration and mass productivity [4–6].

MA materials can be classified into magnetic, dielectric and electric conductive categories according to the absorption mechanisms [1, 7, 8]. The most widely used MA materials are based on magnetic loss strategy, for example Fe_3_O_4_ and ferrite. These magnetic materials show good MA performance, whereas high filler ratio, high density and low corrosion resistance limit their applications. Magnetic materials with filler ratios higher than 50 wt% were frequently reported [9–12]. In such cases, the mechanical properties and dimensional stability of the bulk materials were severely degraded. On the other hand, carbonaceous materials show advantages of low density and low filler content [13, 14]. Among all candidates, graphene has shown pronounced potential owing to its high surface area, tunable electrical conductivity, low density, high stability and good processibility [15–18]. Unfortunately, the MA performance of graphene still remains in a relatively low level. Singh et al. [19] reported the best MA capability of pure graphene by compounding reduced graphene oxide (RGO) with rubber. The composite displays an efficient absorption bandwidth (EAB) of 4.5 GHz, which is moderate among MA materials. Very recently, Chen et al. [20–22] reported that graphene aerogel could act as a good microwave attenuation material with a high EAB (up to 60.5 GHz measured by arch method, around 8 GHz measured by transmission line method) owing to the 3D porous network. Besides, many efforts have been devoted to compound graphene with inorganic magnetic matters for graphene-based MA materials. Zhang et al. [23] designed the RGO/MnFe_2_O_4_ composite with an EAB of 4.88 GHz and a minimum reflection loss (RL) of −30 dB. Feng et al. [24] coated ZnFe_2_O_4_ with SiO_2_ and RGO, which exhibited an EAB of 6 GHz and a minimum RL of -43.9 dB. However, no attention has been paid to the microstructure design of individual graphene, which might have a great impact upon the MA performance. Compared with layered structure, folded graphene assemblies with high porosity can induce the multi-reflection loss of microwave [25].

Herein, we fabricated porous Gmfs powder with high MA performance. Folded graphene sheets assemble together into flower-shaped microparticles, forming a skeleton structure with a high surface area of 230 m^2^ g^−1^ and a low density of 40–50 mg cm^−3^. The maximum EAB reaches 5.59 GHz and the minimum RL is up to -42.9 dB, which is higher than pure graphene fillers and most graphene-based materials in the literature. Moreover, the low filler content (10 wt%) indicates high cost efficiency, benefiting practical applications. The excellent MA ability of Gmfs is attributed to the skeleton microstructure, which can not only promote the attenuation of microwave by multi-reflection between graphene layers, but also favor the formation of conductive network.

## Experimental Section

### Materials

Aqueous suspension of single-layer graphene oxide (GO) with a thickness of 0.8 nm and average lateral sizes of 40–50 μm was commercially available from Gaoxitech (http://www.gaoxitech.com/). All reagents were purchased from Sinopharm from Chemical Reagent Co., Ltd., and used as received. Commercialized graphene powder (CG) was purchased from Asfour (http://www.asfour.com.tw/).

### Preparation of Flower-Shaped GO

Flower-shaped GO (fGO) was prepared via a spray-drying procedure. The obtained 4 mg g^−1^ GO aqueous dispersion was nebulized into small droplets, which were carried by heated air under 140 °C. The water evaporated in a few seconds, leading to the crumpling and folding of GO sheets. The nozzle was in two-fluid mode with the diameter of 400 μm. The dried fGO was collected in a cyclone separator.

### Pre-Reduction and Thermal Annealing of fGO

One gram of fGO was sealed in a glass pot with the addition of 5 drops of hydrazine. The glass pot was kept in the 80 °C oven for 12 h. The obtained reduced fGO turned from yellow to black. Afterward, the reduced fGO was annealed in the tube furnace. The temperature increased from room temperature to 1300 °C in 160 min and kept under that temperature for 1 h to get Gmfs. The protective gas was nitrogen.

### Thermal Annealing of CG

CG was annealed in a tube furnace, following the same procedure of fGO.

### Characterization

The morphologies of the samples were obtained from a Hitachi S4800 field emission scanning electron microscopy (SEM) system. High-resolution transmission electron microscopy (HRTEM) images were collected on a JEM-2010 HRTEM with an accelerating voltage of 120 kV. Nitrogen cryoadsorption was measured on AUTOSORB-IQ-MP (Quantachrome Inc., USA). All samples were outgassed under 120 °C for 1 h. Raman spectra were recorded on a Labram HRUV spectrometer operating at 514 nm. X-ray diffraction (XRD) pattern was measured with an X’Pert Pro (PANalytical) diffractometer using monochromatic Cu Kα radiation (λ = 1.5406 Å) at 40 kV. X-ray photoelectron spectroscopy (XPS) studies were carried out using a PHI 5000C ESCA system operated at 14.0 kV. All binding energies were referenced to the C 1s neutral carbon peak at 284.8 eV. Thermogravimetric analyzer (TGA, TA-Q500) was performed from room temperature to 850 at 10 °C min^−1^ heating rate under nitrogen atmosphere. All samples were heated under 120 °C for 15 min to remove the residual water.

### Microwave Absorption Measurement

The complex permittivity was tested via a coaxial line method. Gmfs and CG were mixed with paraffin under different filler contents and shaped as cylindrical toroidal specimens with an outer diameter of 7.0 mm, inner diameter of 3.04 mm and thickness of 4.0 mm. The preparation of the composites was conducted by simply mixing melt paraffin and the fillers with a glass stick. The parameters were obtained from the vector network analyzer. The complex permeability was considered to be that of free space since no ferromagnetic materials were involved. The *RL* of microwave can be calculated as follows:

1 $$Z_{\text{in}} = \sqrt {\frac{{\mu_{r} }}{{\varepsilon_{r} }}} \tanh \left[ {j\left( {\frac{2\pi }{c}} \right)fd\sqrt {\mu_{r} \varepsilon_{r} } } \right]$$

2 $$RL({\text{dB}}) = 20\log \left| {\frac{{Z_{\text{in}} - 1}}{{Z_{\text{in}} + 1}}} \right|$$

where *Z*_in_ refers to the normalized input impedance of a metal-backed microwave absorbing layer, *μ*_*r*_ and *ε*_*r*_ represent the complex permeability and complex permittivity, respectively, *f* is the frequency of microwaves, *d* is the thickness of the absorber and *c* is the velocity of light in free space.

### Electric Conductivity Measurement

The conductivities of all samples were measured on an electrochemical workstation (CHI660e, CH Instruments, Inc.). According to the percolation theory, the electric conductivity follows the power law: *δ* *∝* *(φ* − *φ*_c_ *)* ^*t*^ [26], where *φ*_*c*_ is the percolation volume fraction and *t* is the critical exponent. *φ*_*c*_ can be obtained by fitting the logδ–*φ* curves. The further fitting of logδ versus log(*φ* − *φ*_*c*_) gives *t*. The transformation between *φ* and weight fraction (*w*) follows: *φ* = wρ_*m*_/(wρ_*m*_ + ρ_*f*_), where ρ_*m*_ and ρ_*f*_ are the density of matrix and filler, respectively. Here ρ_*m*_ is the density of paraffin (0.9 g cm^−3^) and ρ_*f*_ is the density of graphene (2.2 g cm^−3^).

## Results and Discussion

**Fig. 1:**
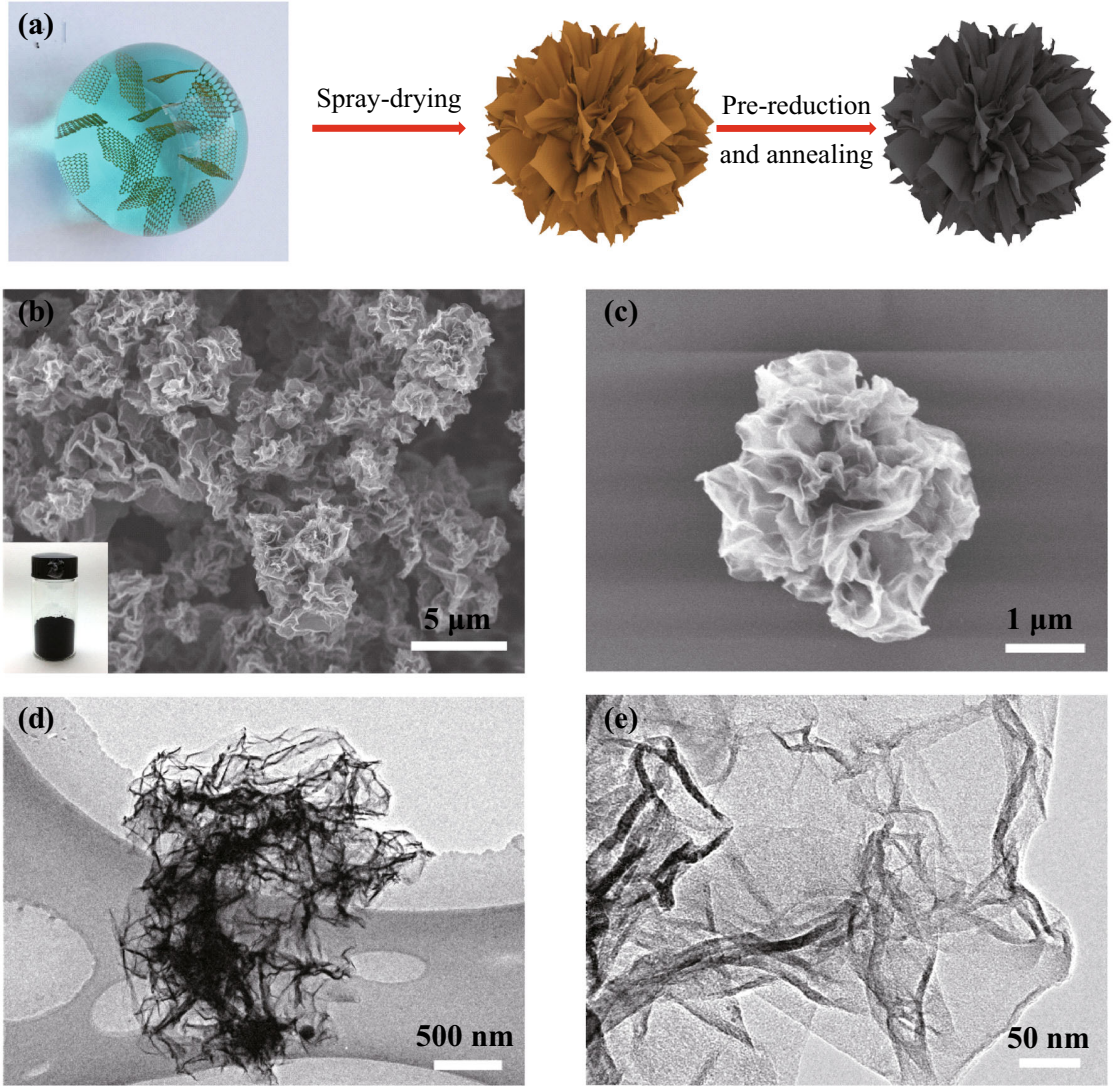
**a** Schematic illustration of the formation of Gmfs. **b**, **c** SEM images of Gmfs. The insert is the digital picture of the 0.4 g Gmfs powder in a 20-mL bottle. **d**, **e** TEM images of an individual Gmfs

As illustrated in Fig. 1a, Gmfs were prepared via a three-step protocol, including spray drying of GO aqueous dispersion, chemical reduction and thermal annealing at 1300 °C. During the spray-drying process, aqueous dispersion of GO was nebulized into microsized droplets and carried by heated air (140 °C) [27]. The capillary force induced by immediate evaporation of water folded GO sheets into a highly crumpled structure, giving rise to flower-shaped GO (fGO) [28, 29]. Chemical reduction was conducted to mildly reduce fGO and preserve the folded microstructures (Fig. S1). The subsequent thermal annealing eliminated the remaining functional groups to restore the electric conductivity of graphene [30, 31].

The obtained Gmfs powder is black and shows a low tap density of 40–50 mg cm^−3^ (the insert in Fig. 1b). As revealed in the SEM images (Fig. 1b, c), the highly folded graphene sheets assemble into microflowers with a size of 2–5 μm. A closer observation of a single Gmf exhibits that there are voids between rippled graphene layers. According to TEM observations, the graphene sheets fold toward the core of Gmfs and form ridges, corresponding to the dark areas in Fig. 1d. Besides, there are plenty of wrinkles on graphene sheets (Fig. 1e). The corrugation of graphene layers hinders the stacking of graphene and enhances the porosity in the microstructure. We chose commercial CG powder for comparison, which is composed of multilayered graphene flakes (Fig. S2). Such laminated structure was frequently observed in previous reports on graphene-based MA materials [32, 33]. However, for MA applications, porous structure favors the formation of conductive network and multi-reflection of microwave [13, 34, 35]. Shen et al. [36] reported that the flower-shaped NiO shows higher MA performance compared with stratiform-like and particle-like structures. Thus, the construction of Gmfs may lead to enhanced MA performance compared with CG.

**Fig. 2:**
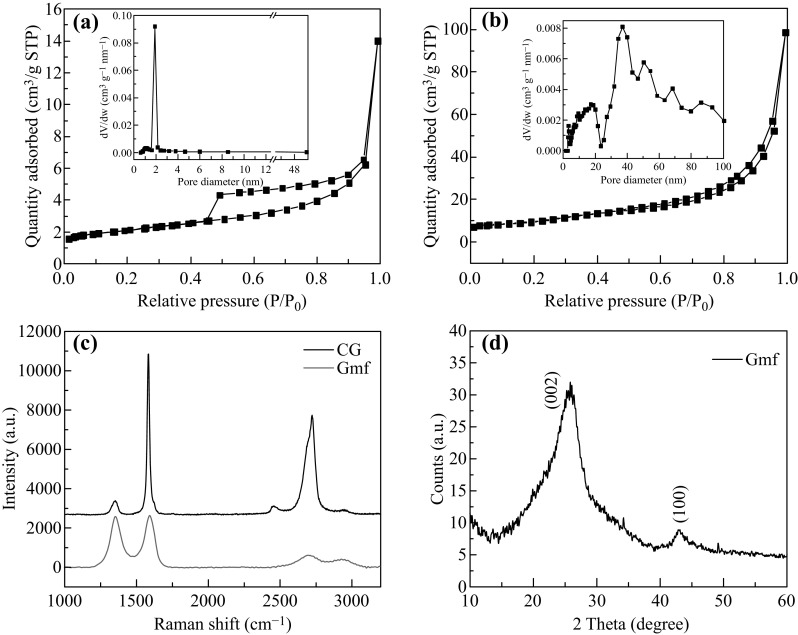
**a** N_2_ absorption/desorption curves of Gmfs. The insert is the calculated pore diameter distribution. **b** N_2_ absorption/desorption curves of CG. The insert is the calculated pore diameter distribution. **c** Raman spectra of Gmfs and CG. **d** XRD pattern of Gmfs

To further reveal the porous microstructure of Gmfs, we conducted the Brunauer–Emmett–Teller (BET) surface area analysis. The absorption/desorption curves and corresponding pore size distributions are shown in Fig. 2a. Gmfs exhibit a type III isothermal curve, and a hysteresis loop can be observed, demonstrating plenty of mesopores in Gmfs. The pore size distribution shows a narrow and intense peak centered at 1.9 nm. The specific surface area and total pore volume of Gmfs are calculated to be 230 and 0.68 cm^3^ g^−1^, respectively, which come from the accessible surface in the voids and tunnels between folded graphene sheets. By contrast, there is no obvious hysteresis loop in the isothermal curve of CG, and the pore size distribution is broad and weak (Fig. 2b). The CG exhibits a low specific surface area of 35.1 m^2^ g^−1^ and a total pore volume of 0.15 cm^3^ g^−1^. Therefore, Gmfs feature a more porous microstructure compared with CG, coinciding with the SEM and TEM observations.

Raman spectroscopy and XRD analysis give more information about the microstructure of Gmfs. As Raman spectrum of Gmfs shown in Fig. 2c, the two intense peaks in the 514 nm are assigned to the D band (1350 cm^−1^) and G band (1580 cm^−1^), respectively [37]. D band represents the defects or *sp* ^3^ carbon in graphene, and G band is related to the graphitic *sp* ^2^ structure. The intensity ratio of the D and G peak is widely used as a metric of disorder in graphene [38]. Thus, the high *I*_D_/*I*_G_ in Gmfs (0.96) demonstrates a more disordered structure compared with CG (*I*_D_/*I*_G_ = 0.14). Such disorder comes from the defects and functional groups left by the reduction in fGO. XRD pattern of Gmfs has one main peak at 26.1°, corresponding to (002) plane (Fig. 2d). This peak is much lower and broader than that of CG, indicating a loose stack of graphene with a lower degree of order (Fig. S3). Both Raman spectroscopy and XRD patterns confirm a skeleton structure with a relatively lower degree of graphitization in Gmfs.

XPS and TGA results verify a high reduction degree in Gmfs. The calculated carbon and oxygen contents of Gmfs are 96.7% and 3.2%, respectively. No obvious oxygen peak can be found in the XPS pattern of Gmfs, indicating the two-step reduction eliminated most functional groups on fGO (Fig. S4a, b). The XPS patterns of Gmfs and CG show little difference, and the element content of CG is similar with that of Gmfs (95.3% carbon and 4.6% oxygen). From the TGA plots, we find that there is negligible weight loss in Gmfs and CG even under 800 °C (Fig. S4c). Therefore, the chemical compositions of Gmfs and CG are nearly identical, attributing to the 1300 °C treatment on both materials. The reduction in GO recovers the graphitic structure of graphene and increases the electrical conductivity, benefiting the microwave attenuation. The defects in Gmfs enhance the polarization effect, which may increase the MA ability.

In order to examine the MA performances of Gmfs, paraffin was selected to make composites for test due to the good processibility and nearly zero reflection loss of microwave. Gmfs can be compounded with paraffin homogeneously without obvious aggregation or precipitation. As shown in Fig. S5a, b, the small crumpled areas in dark color, representing the embedded Gmfs, distribute uniformly on the fracture surface. CG can also form a uniform composite with paraffin, with some graphene flakes stick out from the cross section (Fig. S5c, d). Furthermore, paraffin was dissolved by petroleum ether to evaluate the influence of compounding on the structure of filler materials. It is illustrated that Gmfs keep the flowerlike shape with folded surface after paraffin was washed, and CG is still in multilayered structure (Fig. S6). Therefore, the influence of compounding on microstructures of the graphene fillers is negligible.

**Fig. 3:**
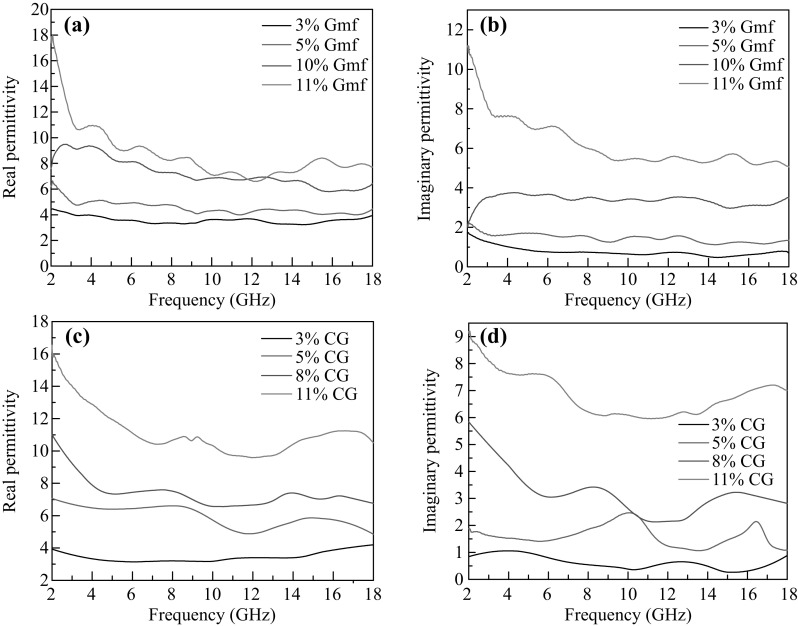
**a** The real parts and **b** imaginary parts of permittivity of Gmfs/paraffin composites with different filler contents. **c** The real parts and **d** imaginary parts of permittivity of CG/paraffin composites with different filler contents

The relative complex permittivity (*ε*) was tested via a coaxial line method. Since there are no magnetic materials in Gmfs, CG or paraffin, the permeability can be regarded as 1. The real parts (*ε*′) and the imaginary parts (*ε*″) of all samples were obtained in the frequency range of 2–18 GHz. As demonstrated in Figs. 3 and S7, both the *ε*′ and *ε*″ decrease with increasing frequency, attributing to the dielectric relaxation [34]. There are significant fluctuations in the curves of CG/paraffin, implying a local inhomogeneity in CG/paraffin composite. Besides, the *ε*′ and *ε*″ increase with the increment of filler content owing to the enhanced polarization and electric conductivity. However, the introduction of Gmfs results in a lower increment in complex permeability compared with CG. We calculated the average *ε*′ and *ε*″ in the frequency range of 8–18 GHz, as summarized in Table 1. The average *ε*′ and *ε*″ of 3 wt% Gmfs/paraffin are in close proximity with those of 3 wt% CG/paraffin. With filling ratio increasing, the *ε*′ of Gmfs/paraffin becomes significantly lower than CG/paraffin. 8 wt% CG/paraffin shows a *ε*′ of 6.96 that is even higher than 10 wt% Gmfs/paraffin. The *ε*″ of Gmfs/paraffin under different filler contents is also lower, except for 8 wt%. For the filler content of 11%, the *ε*′ and *ε*″ of Gmfs/paraffin are 7.56 and 5.44, much lower than those of CG/paraffin. The complex permittivity of carbon materials is determined by the graphitization degree and microstructure [35]. The lower *ε*′ and *ε*″ of Gmfs can be ascribed to the defective porous structure, which will be further discussed below. It is well known that *ε*′ represents the storage capability of the incident electric energy, while *ε*″ influences the dissipation ability based on electromagnetic theory [21]. Microwave cannot be efficiently attenuated when *ε*′ and *ε*″ are low. In contrast, overhigh *ε*′ and *ε*″ lead to the mismatch between air and sample interface. Therefore, the complex permittivity must be optimized to obtain best MA performance.

**Table 1:** Average parameters in the complex permittivities of Gmfs and CG in the frequency range of 8–18 GHz

Filler content (%)	*ε*′ of Gmfs	*ε*″ of Gmfs	*ε*′ of CG	*ε*″ of CG	Tangent loss of Gmfs	Tangent loss of CG
3	3.48	0.65	3.51	0.48	0.19	0.14
5	4.24	1.33	5.58	1.63	0.31	0.29
8	6.51	3.06	6.96	2.78	0.47	0.4
10	6.54	3.31	9.8	6.05	0.52	0.6
11	7.59	5.44	10.46	6.4	0.72	0.61

**Fig. 4:**
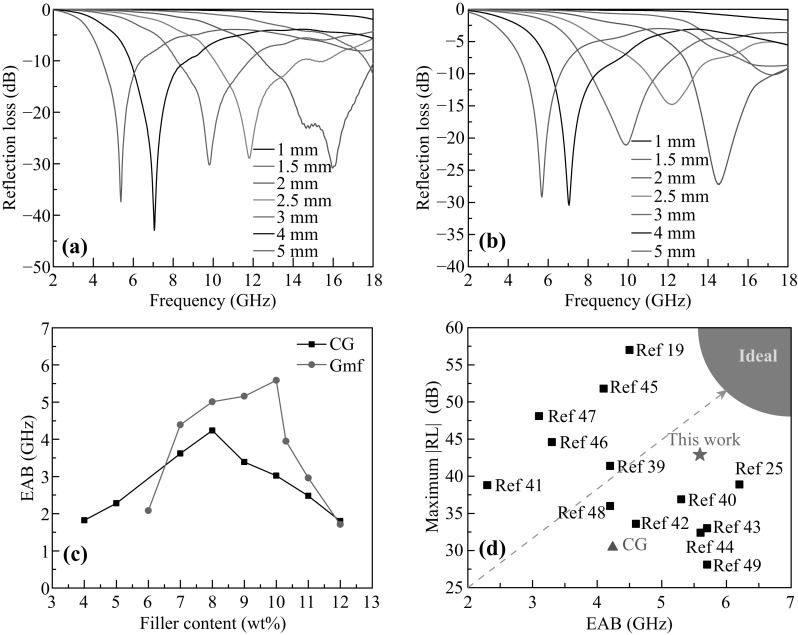
**a** Reflection loss of 10 wt% Gmfs/paraffin composite with various thicknesses. **b** Reflection loss of 8 wt% CG/paraffin composite with various thicknesses. **c** EABs of Gmfs/paraffin and CG/paraffin under different filler contents. **d** Comparison of the maximum |RL| and EAB of Gmf/paraffin and CG/paraffin with reported values in studies

According to the generalized transmission line theory and metal back-panel model, the RL performances of Gmfs/paraffin and CG/paraffin were calculated from the complex permittivity, as shown in Fig. 4a, b. The RL peak shifts to lower frequency as sample thickness increases due to the phenomena of quarter wavelength attenuation in all samples. The optimized filler content for Gmfs is 10 wt%, which is lower than most MA materials. As illustrated in Fig. 4a, the RL can reach −42.8 dB when the thickness is 4 mm. A wide EAB can be obtained as 5.59 GHz (12.41–18 GHz) by setting the thickness as 2 mm; meanwhile, the minimum RL is −30.8 dB. By comparison, 8 wt% is the most rational filler content for CG. The EAB only reaches 4.24 GHz, and the RL remains above -30 dB (Fig. 4b). The EAB values under different filler contents are displayed in Fig. 4c. The calculated EABs of both Gmfs/paraffin and CG/paraffin firstly increase with increasing filler content, attributing to the enhanced storage and dissipation capabilities of the MA fillers, as described in Fig. 3, whereas the MA performance begins to degrade with the addition of excess fillers owing to the dielectric mismatch. Gmfs/paraffin shows wider EABs than CG/paraffin under the same filler contents, verifying the porous microflower structure outperforms multilayered structure for MA. Figure 4d compares the MA performances of Gmfs/paraffin and CG/paraffin with the reported graphene-based nonmagnetic composites in the literature. The broad EAB of Gmfs/paraffin outperforms most materials, accompanied with a high RL value (detailed values are summarized in Table S1) [19, 25, 39–49]. Although high RL values have been achieved in RGO/NBR, RGO/Cu_2_O/Cu/paraffin and PEDOT/graphene/paraffin composites [32, 45, 47], the EAB values keep below 4.5 GHz. Similar EAB is obtained in the CG/paraffin composite (blue triangle in Fig. 4d), implying the MA performances of the graphene-based materials in previous reports might be restricted by the laminated structure. On the contrary, both EAB and RL are enhanced in Gmfs by simply building a skeleton structure of graphene. The EAB of 10 wt% Gmfs covers most Ku band (12–18 GHz), which has been widely used in satellite and radar. Without addition of other materials or doping with chemicals, Gmfs show advantages in facile fabrication, low density and excellent stability.

**Fig. 5:**
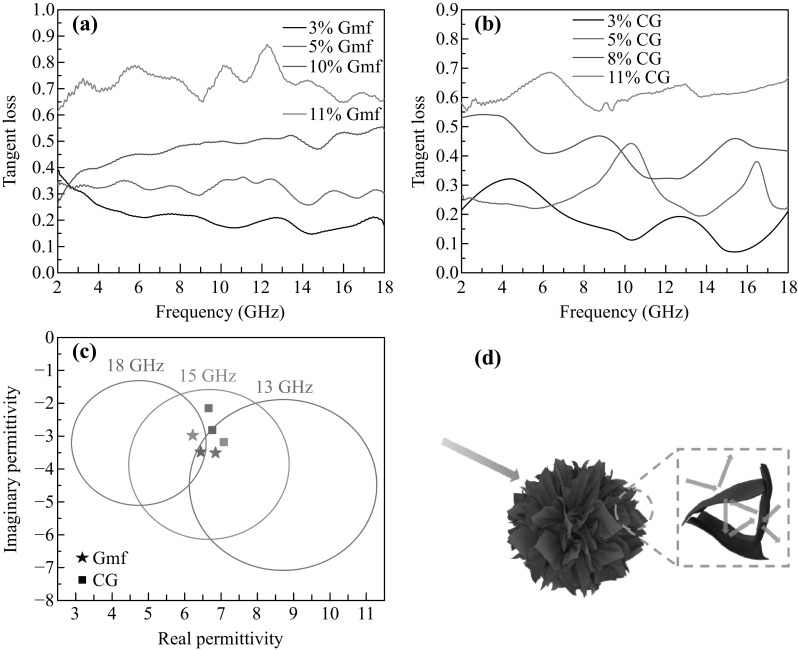
**a** Tangent loss of Gmfs/paraffin under different filler contents. **b** Tangent loss of CG/paraffin under different filler contents. **c** Real parts and imaginary parts of 10 wt% Gmfs/paraffin and 8 wt% CG/paraffin in 13, 15, and 18 GHz. The colored circles represent the rational ranges of *ε*′ and *ε*″ to make RL lower than -10 dB in corresponding frequency. **d** Schematic illustration of MA mechanism of Gmfs

In order to reveal the MA mechanism, the tangent loss (*ε*″/*ε*′) of Gmfs/paraffin and CG/paraffin was compared, as shown in Fig. 5a, b. Tangent loss increases with higher filler content for both Gmfs and CG. The average tangent loss values in the frequency range of 8–18 GHz were calculated and are summarized in Table 1. The addition of Gmfs leads to higher increment in tangent loss, though CG shows greater influence on *ε*′ and *ε*″. The enhanced tangent loss with lower complex permittivity can be attributed to the defective porous microstructure of Gmfs. The reduction in fGO leaves functional groups and defects, which improve the polarization and adjust the conductivity, leading to lower *ε*′ and *ε*″. The skeleton structure generates high surface area, which favors the formation of conductive network and multi-reflection loss. In consequence, Gmfs feature high tangent loss with rational *ε*′ and *ε*″. It is generally accepted that tangent loss represents the capability of converting microwave to other forms of energy. High tangent loss is beneficial for MA, while high values of *ε*′ and *ε*″ are harmful for impedance match. The perfect combination of high tangent loss and reasonable complex permittivity in Gmfs/paraffin gives rise to the high EAB and RL values. Based on the transmission line theory, we can calculate the rational range of *ε*′ and *ε*″ by setting the thickness as 2 mm [50]. As discerned in Fig. 5c, the three circles correspond to the rational ranges of *ε*′ and *ε*″ in 13, 15, and 18 GHz, respectively. RL is below -10 dB when *ε*′ and *ε*″ under corresponding frequency fall into these circles. The complex permittivities of Gmfs/paraffin are in all three circles, indicating the EAB values are below -10 in 13–18 GHz and the EAB is broader than 5 GHz. For CG/paraffin, the complex permittivities are out of the circles of 13 and 18 GHz, leading to a narrow EAB.

The superb MA capability of Gmfs comes from impedance match and effective attenuation in the porous flower-shaped structure. The contribution of the skeleton structure can be divided into three parts. First, the high surface area gives rise to the multi-reflection of microwave [2, 14, 51, 52]. In CG, the inner layers in graphene stacks are blocked by the outer layers, leading to a low utility ratio. Microwave can only be attenuated during the reflection between graphene stacks in CG. By contrast, the highly porous graphene structure with large pore volume promotes the multi-reflection attenuation in Gmfs, as illustrated in Fig. 5d. Second, high porosity is beneficial for the construction of continuous conductive network. As illustrated in Fig. S8a, b, the Gmfs/paraffin gives a percolation threshold of 3.06 wt% and a critical exponent of 3.73. By comparison, CG/paraffin shows a percolation threshold of 3.45 wt% and a critical exponent of 2.75 (Fig. S8c, d). This implies that the highly porous structure of Gmfs favors the formation of conductive network. The absorbed microwave is converted into other forms of energy including electrical and thermal energy through the network [21, 53]. Thus, the porous structure is beneficial to the conversion of microwave. Third, the skeleton structure with defects leads to enhanced polarization [19]. Although the chemical composition of Gmfs is approximately identical with CG, the reduction in fGO results in high disorder, as verified in XRD and Raman analyses. The defects and groups generated after the reduction in fGO induce additional polarization relaxation, as described in previous reports, and the high surface area promotes interfacial polarization.

## Conclusions

We developed a novel strategy to assemble graphene into a highly porous structure, resembling microflowers. The obtained GO dispersion was spray-dried into fGO, followed by chemical reduction and thermal reduction to obtain Gmfs with skeleton microstructure. The combination of porosity and disorder in Gmf gives rise to a broad EAB of 5.59 GHz with a minimum RL of −42.9 dB, which is much higher than those of CG and most graphene-based materials in the literature. The low density (40–50 mg cm^−3^), high specific surface area (230 m^2^ g^−1^) and low filler content (10 wt%) are beneficial to the demands in lightweight and cost reduction. Besides, the facile processing and scalable production of Gmfs are more favorable for practical applications compared with other graphene materials, such as graphene aerogel and graphene composites. Our findings open the way for preparation of high-performance MA materials by rational designing the porous structure.

## Electronic supplementary material

Below is the link to the electronic supplementary material.

Supplementary material 1 (PDF 1056 kb)
