# Supplementary material for: Porous Graphene Microflowers for High-Performance Microwave Absorption
Source: Nanomicro Lett. 2017 Dec 21;10(2):26. doi: 10.1007/s40820-017-0179-8 (PMC6199076; doi:10.1007/s40820-017-0179-8)
Supplement: Supplementary file 1 — Supplementary material 1 (PDF 1056 kb) [file 40820_2017_179_MOESM1_ESM.pdf]

Supporting Information for

Porous Graphene Microflowers for High-Performance Microwave Absorption

Chen Chen[1], Jiabin Xi[1], Erzhen Zhou[2], Li Peng[1], Zichen Chen[2,*], Chao Gao[1,*]

[1]MOE Key Laboratory of Macromolecular Synthesis and Functionalization, Department of Polymer Science and Engineering, Key Laboratory of Adsorption and Separation Materials & Technologies of Zhejiang Province, Zhejiang University, 38 Zheda Road, Hangzhou 310027, People's Republic of China

[2]Department of Mechanical Engineering, Zhejiang University, 38 Zheda Road, Hangzhou 310027, People's Republic of China

*Corresponding authors. E-mail: chaogao@zju.edu.cn, chenzc@zju.edu.cn
Tel: +86-(0)571-87952088

Figures and Table

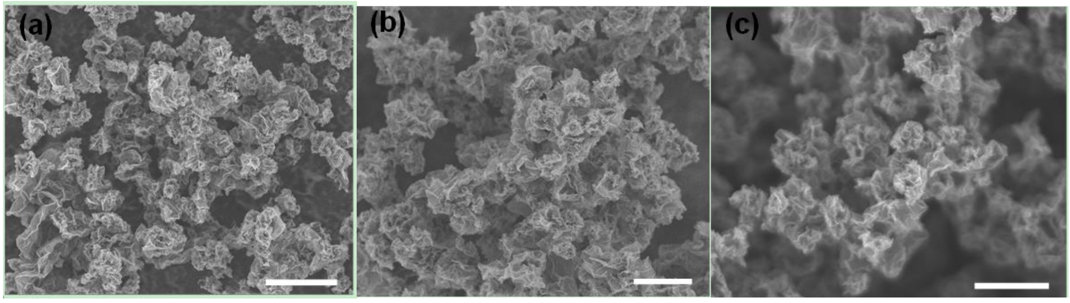

Fig. S1 a SEM image of fGO. **b** SEM image of fGO after chemical reduction. c SEM image of fGO after chemical reduction and thermal treatment. Scale bars: 10 μm in **a**, 5 μm in **b**, **c**

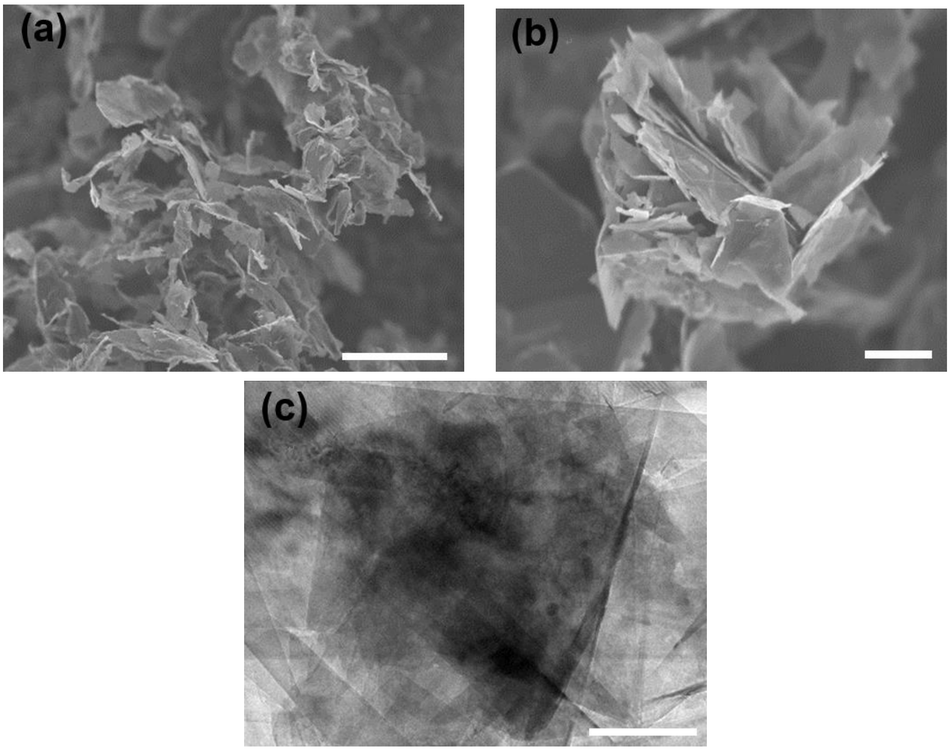

Fig. S2 a, b SEM images of CG. **c** TEM image of CG.
Scale bars: 10 µm in **a**, 2 µm in **b** and 500 nm in **c**

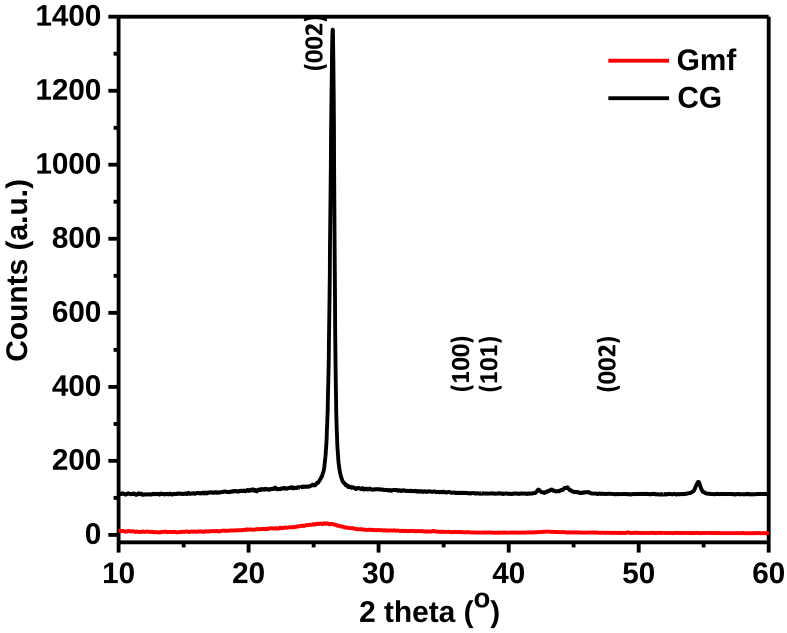

Fig. S3 XRD patterns of Gmf and CG

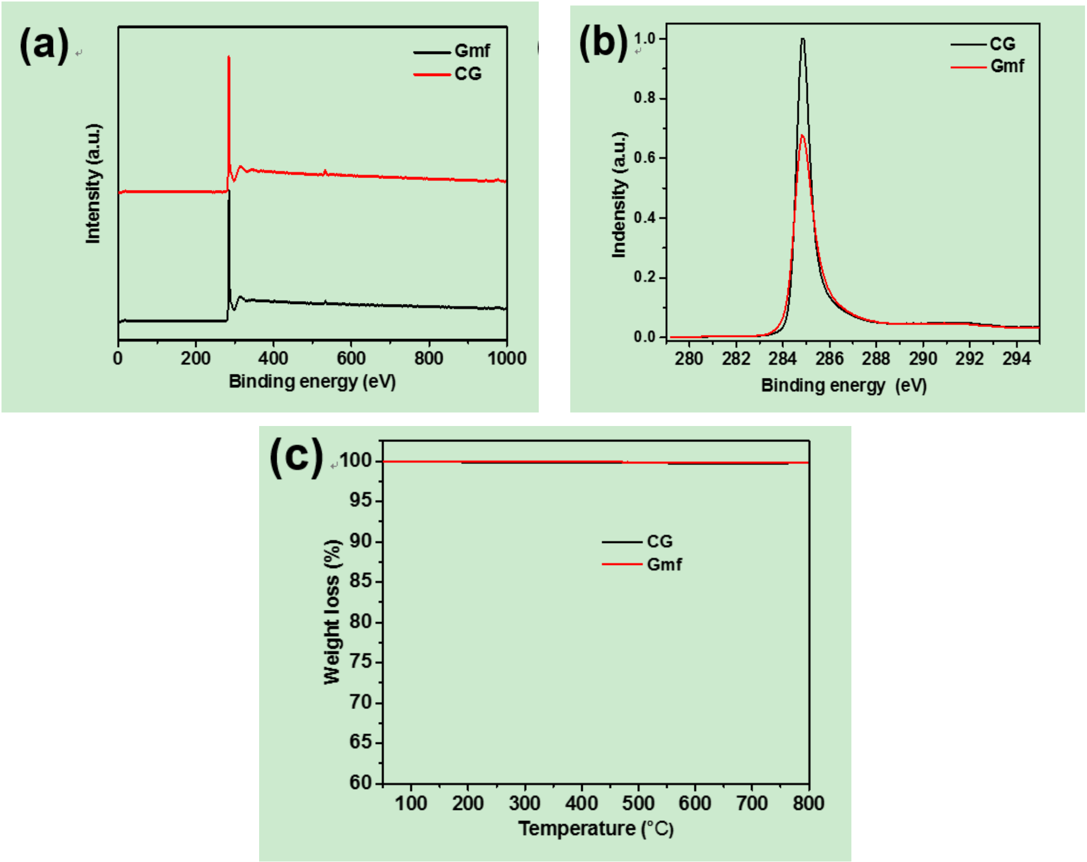

Fig. S4 **a** XPS patterns of Gmf and CG. **b** C 1s spectra of CG and Gmf from XPS analysis. **c** TGA curves of Gmf and CG

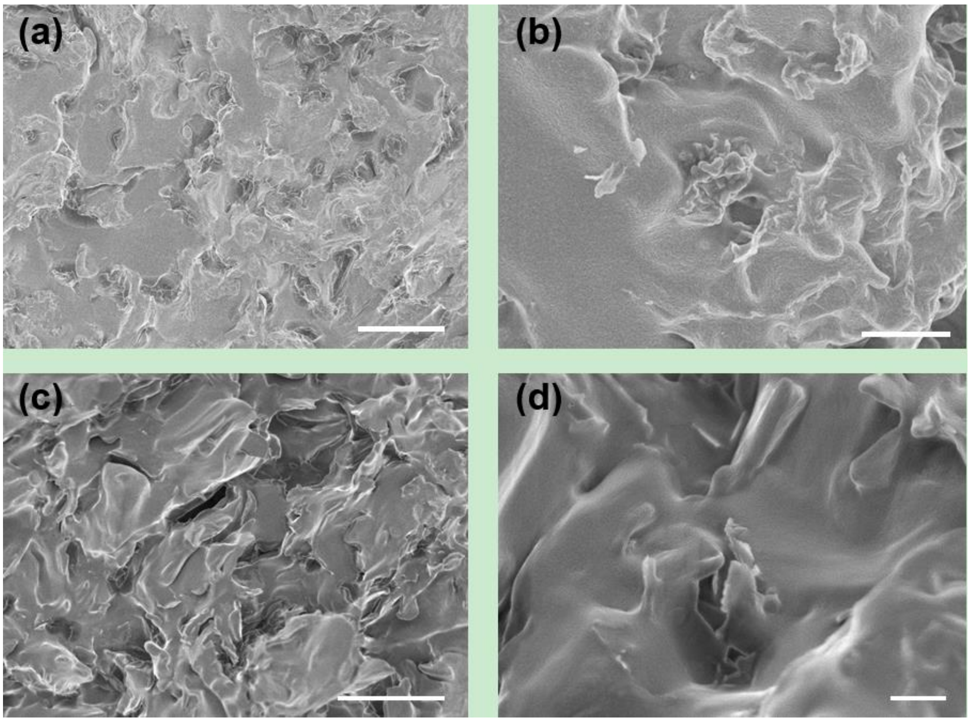

Fig. S5 Cross-section SEM images of Gmf/paraffin **a, b** and CG/paraffin **c, d**. Scale bars: 15 μm in **a, c** and 2 μm in **b, d**

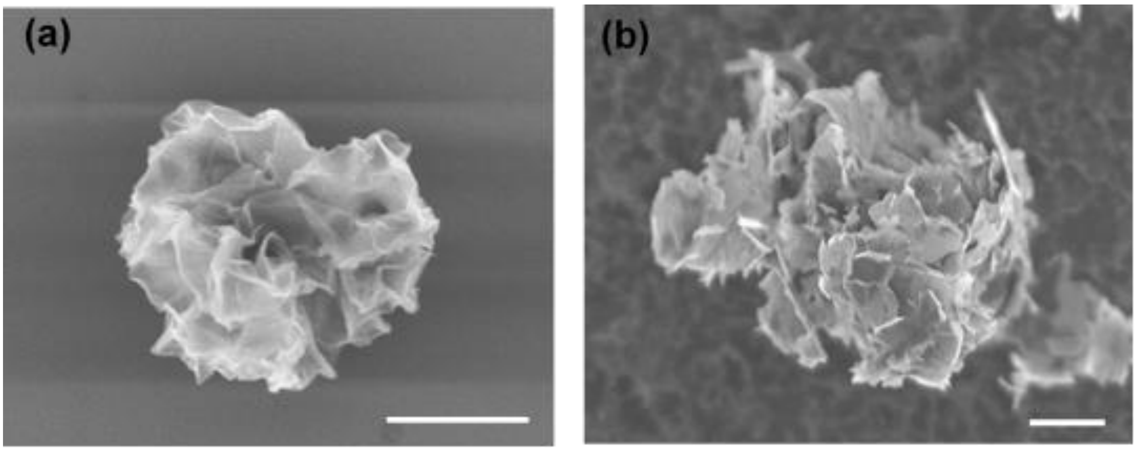

Fig. S6 SEM images of **a** Gmf/paraffin and **b** CG/paraffin composites after paraffin was dissolved by petroleum ether. Scale bars: 1 μm in **a** and 5 μm in **b**

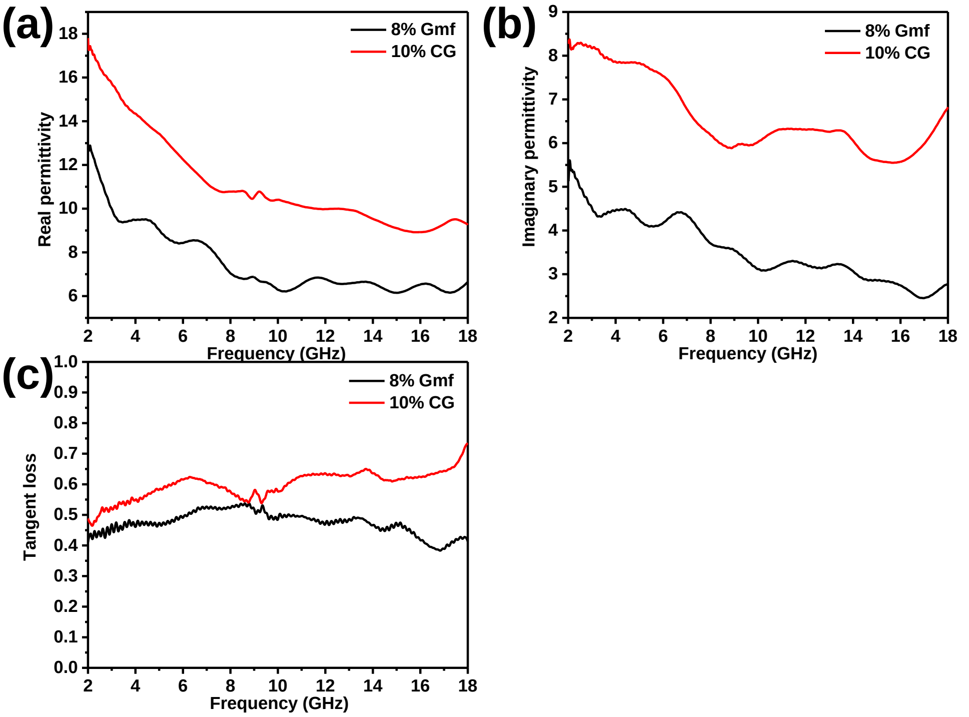

Fig S7 a The real permittivity, **b** imaginary permittivity and **c** tangent loss of 8 wt% Gmf/paraffin and 10 wt% CG/paraffin

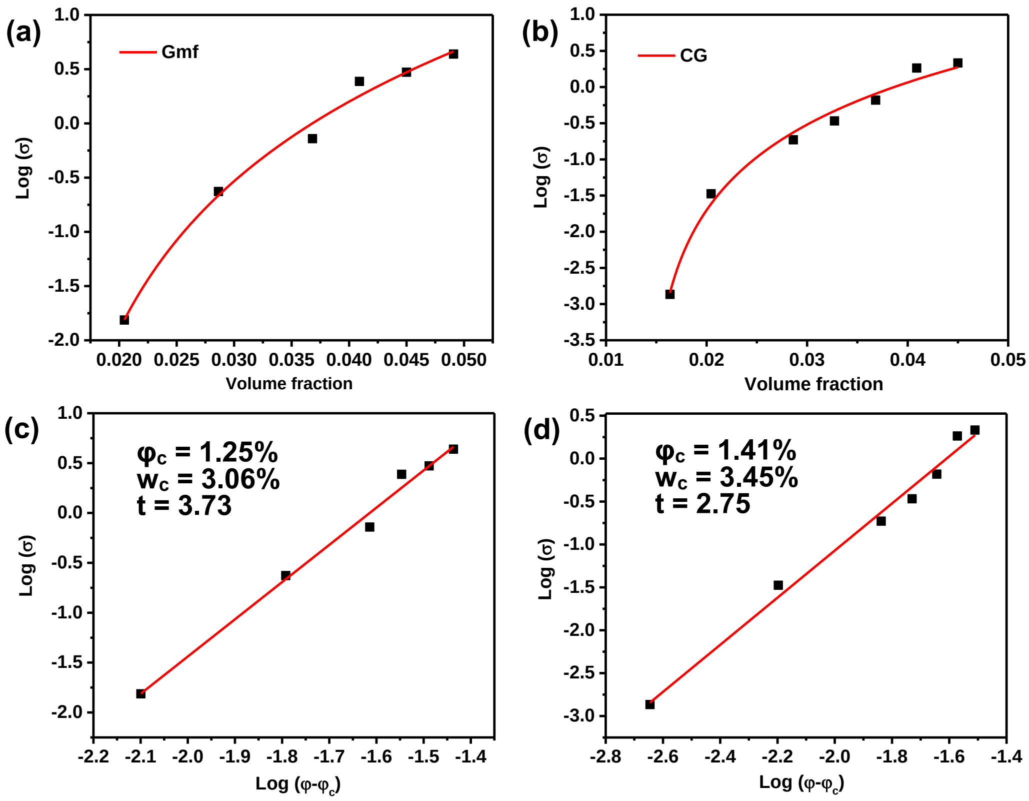

Fig. S8 Log dc conductivity (δ) vs volume fraction (φ) of Gmf/paraffin composites **a** and CG/paraffin composites **b**. Log-log plots of δ vs (φ-φ_c) for Gmf/paraffin composites **c** and CG/paraffin composites **d**. The percolation volume fraction (φ_c), percolation weight fraction (w_c) and critical exponent (t) are shown in the graphs

Table S1 Comparison of the MA performances of Gmf, CG and the reported graphene-based materials

Composition	Thickness (mm)	wt%	Max |RL| (dB)	Frequency range (GHz)	EAB [RL≤ -10] (GHz)	Ref.
Gmf/Paraffin	2	10	42.93	2~18	5.59 (12.41~18)	This work
CG/Paraffin	2	10	29.2	2~18	4.24 (13.14~17.38)	This work
PPy/GO/Paraffin	3	30	38.9	2~18	6.2 (9.2~15.4)	1
GN/Carbon/Paraffin	1.5	10	28.1	2~18	5.7	2
MoS_2-Graphene/Paraffin	2	10	33	2~18	5.7 (11.7~17.4)	3
CR-G/PEO	2	5	32.4	2~18	5.6 (12.4~18)	4
Graphene/PANI/wax	3.5	10	36.9	2~18	5.3(8.2~13.5)	5
B,N-graphene/Paraffin	16	25	33.6	2~18	4.6	6
RGO/NBR	3	10	57	4~12	4.5 (7.5~12)	7
RGO/PANI/Paraffin	2	50	41.4	2~18	4.2(11.7~15.9)	8
Graphene/Silica textile/PF	3.5	76.8	36	8.2~12.4	4.2 (8.2~12.4)	9
RGO/Cu_2O/Cu/Paraffin	1.3	50	51.8	2~18	4.1 (12.1~16.2)	10
Graphene/CNT/Paraffin	3	5	44.6	2~18	3.3 (7.1~10.4)	11
PEDOT/Graphene/Paraffin	2	25	48.1	2~18	3.1 (9.2~12.3)	12
N-graphene/PANI/Paraffin	3	25	38.8	2~18	2.3(6.8~8.8)	13

Reference

[1] X. Chen, J. Chen, F. Meng, L. Shan, M. Jiang, X. Xu, J. Lu, Y. Wang, Z. Zhou, Hierarchical composites of polypyrrole/graphene oxide synthesized by in situ intercalation polymerization for high efficiency and broadband responses of electromagnetic absorption. Compos. Sci. Techno. **127**, 71-78 (2016). https://doi.org/10.1016/j.compscitech.2016.02.033

[2] H. Lv, Y. Guo, Y. Zhao, H. Zhang, B. Zhang, G. Ji, Z.J. Xu, Achieving tunable electromagnetic absorber via graphene/carbon sphere composites. Carbon **110**, 130-137 (2016). https://doi.org/10.1016/j.carbon.2016.09.009

[3] Y. Wang, D. Chen, X. Yin, P. Xu, F. Wu, M. He, Hybrid of MoS_2 and reduced graphene oxide: A lightweight and broadband electromagnetic wave absorber. ACS Appl. Mater. Interfaces **7**(47), 26226-26234 (2015). https://doi.org/10.1021/acsami.5b08410

[4] X. Bai, Y. Zhai, Y. Zhang, Green approach to prepare graphene-based composites with high microwave absorption capacity. J. Phys. Chem. C **115**(23), 11673-11677 (2011). https://doi.org/10.1021/jp202475m

[5] X. Chen, F. Meng, Z. Zhou, X. Tian, L. Shan, et al., One-step synthesis of graphene/polyaniline hybrids by in situ intercalation polymerization and their

electromagnetic properties. Nanoscale **6**(14), 8140-8148 (2014). https://doi.org/10.1039/C4NR01738B

[6] Y. Kang, Z. Chu, D. Zhang, G. Li, Z. Jiang, H. Cheng, X. Li, Incorporate boron and nitrogen into graphene to make BCN hybrid nanosheets with enhanced microwave absorbing properties. Carbon **61**(11), 200-208 (2013). https://doi.org/10.1016/j.carbon.2013.04.085

[7] V.K. Singh, A. Shukla, M.K. Patra, L. Saini, R.K. Jani, S.R. Vadera, N. Kumar, Microwave absorbing properties of a thermally reduced graphene oxide/nitrile butadiene rubber composite. Carbon **50**(6), 2202-2208 (2012). https://doi.org/10.1016/j.carbon.2012.01.033

[8] P. Liu, Y. Huang, Decoration of reduced graphene oxide with polyaniline film and their enhanced microwave absorption properties. J. Polym. Res. **21**(5), 1-5 (2014). https://doi.org/10.1007/s10965-014-0430-7

[9] W.L. Song, X.T. Guan, L.Z. Fan, Y.B. Zhao, W.Q. Cao, C.Y. Wang, M.S. Cao, Strong and thermostable polymeric graphene/silica textile for lightweight practical microwave absorption composites. Carbon **100**, 109-117 (2016). https://doi.org/10.1016/j.carbon.2016.01.002

[10] M. Zong, Y. Huang, H. Wu, Y. Zhao, P. Liu, L. Wang, Facile preparation of RGO/Cu_2O/Cu composite and its excellent microwave absorption properties. Mater. Lett. **109**(1), 112-115 (2013). https://doi.org/10.1016/j.matlet.2013.07.045

[11] L. Wang, Y. Huang, C. Li, J. Chen, X. Sun, A facile one-pot method to synthesize a three-dimensional graphene@carbon nanotube composite as a high-efficiency microwave absorber. Phys. Chem. Chem. Phys. **17**(3), 2228-2234 (2014). https://doi.org/10.1039/C4CP04745A

[12] X. Zhang, Y. Huang, P. Liu, Enhanced electromagnetic wave absorption properties of poly(3,4-ethylenedioxythiophene) nanofiber-decorated graphene sheets by non-covalent interactions. Nano-Micro Lett. **8**(2), 131-136 (2016). https://doi.org/10.1007/s40820-015-0067-z

[13] L. Wang, Y. Huang, H. Huang, N-doped graphene@polyaniline nanorod arrays hierarchical structures: Synthesis and enhanced electromagnetic absorption properties. Mater. Lett. **124**(6), 89-92 (2014). https://doi.org/10.1016/j.matlet.2014.03.066
